# Interplay between Caveolin-1 and body and tumor size affects clinical outcomes in breast cancer

**DOI:** 10.1016/j.tranon.2022.101464

**Published:** 2022-06-01

**Authors:** Christopher Godina, Vineesh Indira Chandran, Magdalena Barbachowska, Helga Tryggvadottir, Björn Nodin, Edward Visse, Signe Borgquist, Karin Jirström, Karolin Isaksson, Ana Bosch, Mattias Belting, Helena Jernström

**Affiliations:** a Division of Oncology, Department of Clinical Sciences in Lund, Lund University and Skåne University Hospital, Barngatan 4, Lund SE-221 85, Sweden; b Department of Hematology, Oncology and Radiation Physics, Skåne University Hospital, Sweden; c Division of Oncology and Therapeutic Pathology, Department of Clinical Sciences in Lund, Lund University and Skåne University Hospital, Lund, Sweden; d Division of Neurosurgery, Department of Clinical Sciences in Lund, Lund University and Skåne University Hospital, Lund, Sweden; e Department of Oncology, Aarhus University and Aarhus University Hospital, Aarhus, Denmark; f Division of Surgery, Department of Clinical Sciences in Lund, Lund University and Kristianstad Hospital, Kristianstad, Sweden; g Department of Immunology, Genetics and Pathology, Science for Life Laboratory, Uppsala University, Uppsala, Sweden

**Keywords:** Breast cancer, Caveolin-1, Anthropometrics, Cohort, Prognosis, ALNI, Axillary lymph node involvement, BRCA, Breast cancer gene, BCFI, Breast cancer-free interval, BMI, Body mass index, CAF, Cancer-associated fibroblast, CAV1, Caveolin-1, CBCFI, Contralateral breast cancer-free interval, DMFI, Distant metastasis-free interval, EGFR, Epidermal growth factor receptor, EMT, Epithelial-mesenchymal transition, ER, Estrogen receptor, GOBO, Gene expression-based Outcome for Breast cancer, HER2, Human epidermal growth factor receptor 2, HIF1, Hypoxia-induced factor 1, HR, Hazard ratio, IGF, Insulin-like growth factor, IHC, Immunohistochemistry, LRFI, Locoregional recurrence-free interval, mRNA, Messenger ribonucleic acid, NDRFI, Non-distant recurrence-free interval, NF-κB, Nuclear factor kappa-light-chain-enhancer of activated B cells, NoE, Number of events, OS, Overall survival, PR, Progesterone receptor, REMARK, Reporting recommendations for tumor marker prognostic studies, RPPA, Reverse phase protein array, STRING, Search Tool for the Retrieval of Interacting Genes/Proteins, TBSAS, Time between surgery and staining, TCGA, The Cancer Genome Atlas, TGFβ, Transforming growth factor-beta, TMA, Tissue microarray, TME, Tumor microenvironment, TNBC, Triple-negative breast cancer

## Abstract

**Background:**

Caveolin-1 (CAV1) is associated with cholesterol-rich membrane raft domains and is a master regulator of cell signaling and membrane transport. Here, we investigated CAV1’s role in cellular compartments of breast cancer in relation to signaling pathways, clinicopathological features, and clinical outcomes.

**Methods:**

CAV1 levels were evaluated with immunohistochemistry in cytoplasm of invasive tumor cells and stromal cells in tumor tissue microarrays from a cohort of 1018 breast cancer patients (inclusion 2002–2012, Sweden). Cytoplasmic and stromal CAV1 were categorized as positive/negative and strong/not strong, respectively. CAV1 expression in relation to clinical outcomes was assessed with Cox regression. Investigations into CAV1 functional pathways was conducted in the STRING, GOBO, and TCGA databases.

**Results:**

CAV1 expression was associated with non-luminal subtypes, cell cycle control, inflammation, epithelial-mesenchymal transition, and the IGF/Insulin system. Generally, CAV1 was not associated with recurrence risk. Stromal CAV1’s impact on recurrence risk was modified by BMI ≥25 kg/m^2^ (*P*_interaction_ = 0.002), waist ≥80 cm (*P*_interaction_ = 0.005), and invasive tumor size (pT2/3/4) (*P*_interaction_ = 0.028). In low-risk patients only, strong stromal CAV1 significantly increased recurrence risk (HRs_adj_ ≥1.61). In all patients, positive cytoplasmic CAV1 conferred >2-fold risk for contralateral disease HR_adj_ 2.63 (95% CI 1.36–5.10). Strong stromal CAV1 conferred nearly 2-fold risk for locoregional recurrence HR_adj_ 1.88 (95% CI 1.09–3.24).

**Conclusions:**

CAV1’s prognostic impact depended on its localization, anthropometric, and tumor factors. Stromal CAV1 predicted high recurrence risk in a group of supposedly ‘low-risk’ patients. Cytoplasmic CAV1 predicted metachronous contralateral disease. If confirmed, CAV1 could be used as treatment target and for risk-stratification.

## Highlights

• Breast tumor CAV1 levels were assessed in relation to phenotype and prognosis.
• CAV1’s prognostic impact depended on anthropometric and tumor factors.
• Stromal CAV1 predicted high recurrence risk in ‘low-risk’ patients.
• Stromal CAV1 nearly doubled locoregional recurrence risk in breast cancer.
• Cytoplasmic CAV1 was a marker for metachronous contralateral breast cancer.

## Background

Caveolin-1 (CAV1) is a protein located in cholesterol-rich plasma membrane raft domains, defined as caveolae, and functions as a master regulator of cell signaling and transport [1,2]. CAV1 modulates many cellular functions, including nutrient and drug internalization, tumor-stroma interactions, hypoxia response, inflammation, epithelial-mesenchymal transition (EMT), and cell cycle regulation [1], [2], [3]. CAV1 and caveolae have been implicated in cancer cell metabolic regulation, including mitochondrial bioenergetics and fatty acid metabolism [4]. Many of these cellular functions, which are important drivers of breast cancer aggressiveness, lack established biomarkers and targets [3,5]. This fact highlights the need to investigate new potentially relevant biomarkers, such as CAV1.

CAV1 is also expressed in the stromal compartment and can be considered a marker of the tumor microenvironment (TME) [3]. The importance of TME for tumor development, metastasis, and treatment resistance is increasingly recognized [6]. The TME may also link the host and the tumor, particularly for adiposity and metabolic-related effects. Different CAV1 genotypes were associated with metabolic and obesity-related factors, such as waist circumference [7]. Challenges remain in elucidating the role of CAV1 in the TME comprised of several distinct cell types, including immune cells, cancer-associated fibroblasts (CAF), endothelial cells, and adipocytes with different functions [8]. The TME's function depends on interactions with the tumor cells [9,10]. Therefore, there is a need for new markers to investigate how the TME combined with established prognostic markers might modulate prognosis and treatment prediction.

Despite compelling *in vitro* data, the clinical significance of CAV1 remains unclear, and smaller studies investigating its prognostic impact in breast cancer have provided conflicting results [11], [12], [13]. Since breast cancer is a heterogeneous disease with different tumor biology, receptor expression, and outcomes [14], [15], [16], CAV1’s prognostic impact may be context-dependent. Loss of CAV1 in stroma indicated transformation of surrounding tissue into TME through tumor cell and stroma interactions mediated by various signaling pathways, including TGFβ in early breast cancer [1,3]. Conversely, other works have reported that upregulation of CAV1 in TME promotes invasion and metastasis at a later stage in breast cancer development [17].

The interplay between tumor size and stromal CAV1 is yet to be explored. CAV1 is rarely expressed in luminal cells in normal breast tissue but rather in myoepithelial cells [18,19]. In breast cancer, CAV1 interacts with both HER2 and ER [20], suggesting that CAV1 plays different roles depending on receptor expression and localization. Thus, we investigated the role of CAV1 in signaling pathways and different cellular compartments of breast cancer in relation to clinical outcomes overall and different patient subgroups.

## Materials and methods

### Cohort description

**Fig. 1:**
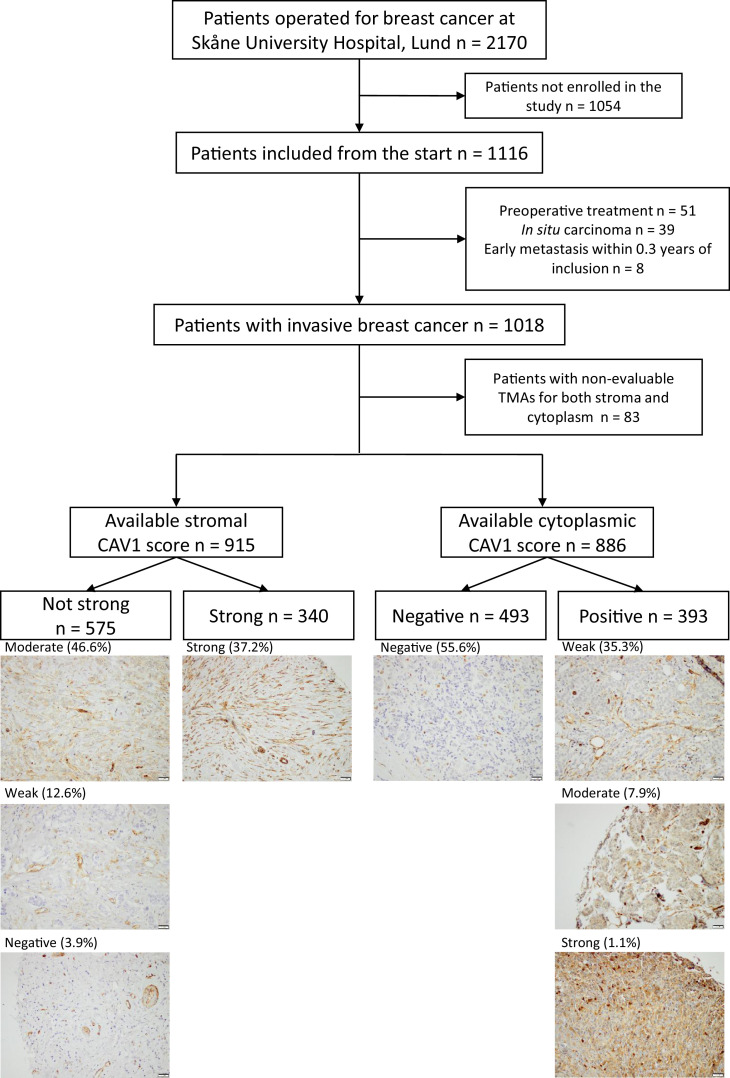
Flowchart of included and excluded patients and representative pictures of each CAV1 staining category in stroma and cytoplasm.

The BCblood cohort is a population-based cohort consisting of primary breast cancer patients operated at Skåne University Hospital in Lund. Ethical approval has been granted by the Lund University Ethics Committee (Dnr 75-02, Dnr 37-08, Dnr 658-09, and amendments). All participants provided written informed consent. The cohort has been described in detail elsewhere [21,22]. In short, patients diagnosed with a first breast cancer and no other malignancies within 10 years before inclusion, before surgery, were included. At inclusion, the participants answered a questionnaire regarding lifestyle and anthropometric measurements were taken by research nurses. Medical records and registries were used to obtain clinical data. Exclusion criteria were carcinoma *in situ*, preoperative treatment, and distant metastasis within 0.3 years of inclusion. A final number of 1018 patients included October 2002 to June 2012, remained (Fig. 1). The patients were followed until June 30, 2019.

Most patients included before November 2005 had missing HER2 status. HER2 status for patients with missing status was obtained from dual gene protein staining of HER2 on the TMAs and showed 97.7% agreement with pathological assessment [23]. Following the Swedish clinical routine, the ER and PR positivity cut-offs were >10% stained nuclei. Anthropometric factors were dichotomized as follows, BMI ≥25 kg/m^2^, waist ≥80 cm, and breast volume ≥850 ml [24].

### TMA construction, staining, and evaluation

CAV1 staining on TMAs was performed as previously described [21,25]. In brief, duplicate 1 mm cores were stained with a primary rabbit polyclonal CAV1 antibody (diluted 1:1000; ab2910, Abcam, Cambridge, UK). Two evaluators (V.I.C. and M.Ba.) blinded to clinical data evaluated CAV1 as previously described [25]. Both stainings of the cytoplasm of invasive tumor cells and stromal cells were dichotomized: cytoplasm as positive (1+/2+/3+) or negative (0) and stroma as strong (3+) or not strong (0/1+/2+). The stromal and cytoplasmic categories were combined to create a joint cytoplasmic/stromal CAV1 status with four categories: negative/not strong, negative/strong, positive/not strong, and positive/strong (representative images in Fig. 1). There were 19 patients with bilateral invasive tumors. Scoring of both tumors was possible for ten patients. The highest category was used for the four cases where the categories differed. Clinicopathological information was collected from the corresponding side.

### TCGA dataset

Gene-level RNA-sequence and reverse-phase protein array (RPPA) data for CAV1, other proteins involved in key signaling pathways in breast cancer [26], and corresponding clinical data were obtained and processed, as previously described from a subcohort of 809 patients [27] of TCGA (https://portal.gdc.cancer.gov).

### Statistical analyzes

For statistical analyzes, STATA® version 17.0 (StataCorp, College Station, TX, US) was used. Mann-Whitney U and Kruskal-Wallis tests were used to determine whether categories of cytoplasmic, stromal, and combined CAV1 status differed according to time between surgery and staining (TBSAS). Both stromal and combined CAV1 status were negatively associated with TBSAS (both *P* < 0.001). Therefore, TBSAS was always included as a covariate in multivariable models, including stromal or combined CAV1. Cytoplasmic, stromal, and combined CAV1 in relation to patient and tumor characteristics were analyzed using logistic regression (simple or multinomial) adjusted for age at inclusion (continuous). The negative/not strong CAV1 status was used as reference.

Three main endpoints were used for survival; any first breast cancer recurrence, distant metastasis, and death. During exploratory analyzes, further survival analyzes were conducted for locoregional recurrence, contralateral breast cancer, and non-distant recurrence. Breast cancer-free interval (BCFI), locoregional recurrence-free interval (LRFI), contralateral breast cancer-free interval (CBCFI), non-distant recurrence-free interval (NDRFI), and distant metastasis-free interval (DMFI) were defined as the time from inclusion until the first event. Patients without recurrences were censored at the time of the last follow-up before emigration, death, or last follow-up by June 30, 2019. Overall survival (OS) was defined as the time until death or last follow-up by June 30, 2019.

Univariable survival analyzes were conducted with Kaplan-Meier curves and Log-rank tests. Cox proportional hazards models were used for multivariable survival analyzes. Two models were used. Model 1 was adjusted for age and tumor characteristics. Model 2 included model 1 and was further adjusted for postoperative treatments before any event. Schoenfeld's residuals were used to test the proportional hazard assumption for stromal, cytoplasmic, and combined CAV1 status in model 2 for the three main endpoints. Survival analyzes with CBCFI as an endpoint were restricted to patients without bilateral tumors.

To examine potential effect modifications by anthropometric factors and tumor characteristics on the associations between cytoplasmic, stromal, and combined CAV1 and BCFI, DMFI, and OS, formal two-way interaction analyzes were performed in model 1. For adjuvant treatments, formal two-way interaction analyzes were performed in model 2. The interaction analyzes for tamoxifen and aromatase inhibitors were restricted to patients with ER^+^tumors.

### Missing data

Most (931, >99%) patients included in the survival analyzes had no missing values. However, some had missing data on BMI (*n* = 27) and HER2 (*n* = 34; 93% complete cases). Based on the pattern of missing data, the missing values were assumed to be ‘missing at random’. Therefore, missing values for all variables in the survival analyzes, including BMI and HER2, were imputed using chained equations. One-hundred datasets, including 935 patients, were created from ten iterations each. Pooled results of the multiple imputation were used for the survival analyzes with further adjustment for BMI ≥25 kg/m^2^ and HER2.

In the TCGA database, correlations were assessed using Spearman's rank (R_s_) for all patients. No survival analyzes were conducted due to scarce follow-up (median follow-up 1.3 years for patients at risk). Also, *CAV1* mRNA expression was investigated in a panel of 51 human breast cancer cell lines and the tumors of 1881 patients in the Gene expression-based Outcome for Breast cancer (GOBO) platform [28,29]. The cell lines were classified according to Neve et al. [30]. Moreover, functional protein associations for CAV1 were explored in the STRING database [31].

Power calculations were performed using PS Power and Sample Size Calculation program version 3.1.16 (Vanderbilt University, TN, USA) [32]. For the power calculation, we assumed that with 855 patients (380 with positive cytoplasmic staining and 330 with strong stromal staining), a 10-year accrual time with an additional 7-year follow-up, true HRs of ≤0.785 or ≥1.299 and ≤0.781 or ≥1.306, respectively, would be detectable with 80% power and α of 0.05.

The REporting recommendations for Tumor MARKer prognostic studies (REMARK) were followed [33]. *P*-values were considered as the level of evidence against the null hypothesis, and all *P*-values are two-tailed. Nominal *P*-values are presented without adjustment for multiple testing due to the exploratory nature of this study [34].

## Results

### Patient characteristics in relation to CAV1 levels

Table 1 (cytoplasm/stroma) and Supplementary Table 1 (combined) presents the patients’ characteristics in relation to CAV1 levels. Positive cytoplasmic CAV1 was associated with anthropometric factors related to a poor metabolic profile, such as large breast volumes (*P*_adj_ = 0.016) and somewhat larger waists (*P*_adj_ = 0.092). Strong stromal CAV1 was associated with younger age (*P*_adj_ < 0.001) and more hormonal intrauterine device use (*P*_adj_ = 0.039). Likewise, both negative/strong and positive/strong CAV1 status were associated with younger age (both *P*_adj_ ≤ 0.009). CAV1 positive/not strong showed stronger associations with large breast volumes (*P*_adj_ = 0.003) and waists (*P*_adj_ = 0.058) than positive cytoplasmic CAV1, irrespective of stromal CAV1.

**Table 1:** CAV1 in cytoplasm and stroma in relation to patient and tumor characteristics.

	All patients	Missing	CAV1 cytoplasm *n* = 886		CAV1 stroma *n* = 915		Patients with non-evaluable TMAs	
			Negative	Positive	Not strong	Strong	CAV1 cytoplasmic	CAV1 Stroma
	*n* = 1018		*n* = 493 (55.6%)	*n* = 393 (44.4%)	*n* = 575 (62.8%)	*n* = 340 (37.2%)	*n* = 132	*n* = 103
	Number (%)		Number (%)	Number (%)	Number (%)	Number (%)	Number (%)	Number (%)
	or Median (IQR)		or Median (IQR)	or Median (IQR)	or Median (IQR)	or Median (IQR)	or Median (IQR)	or Median (IQR)
Age at inclusion, years	61.1 (52.1–68.1)	0	60.9 (52.7–68.4)	61.3 (51.2–67.7)	61.8 (53.9–68.4)	58.7 (50.0–67.4)	61.1 (51.6–68.3)	63.3 (54.1–70.2)
BMI ≥25 kg/m^2^	503 (50.8)	28	240 (50.1)	196 (51.4)	283 (50.2)	160 (49.4)	67 (51.5)	60 (58.8)
Waist circumference ≥80 cm	731 (74.6)	38	345 (72.5)	289 (77.1)	414 (74.3)	240 (74.3)	97 (75.2)	77 (77.0)
Breast volume ≥850 ml	492 (57.3)	160	225 (53.4)	201 (61.3)	272 (56.3)	166 (57.8)	66 (60.6)	54 (61.4)
Alcohol abstainer, yes	106 (10.4)	3	53 (10.8)	41 (10.5)	57 (10.0)	42 (12.4)	12 (9.1)	7 (6.8)
Preoperative smoker, yes	206 (20.3)	2	98 (19.9)	76 (19.4)	111 (19.3)	71 (20.9)	32 (24.2)	24 (23.3)
Coffee, ≥2 cups/day	824 (80.9)	0	391 (79.3)	324 (82.4)	483 (84.0)	262 (77.1)	109 (82.6)	79 (76.7)
Oral contraceptives, ever	722 (71.0)	1	354 (72.0)	274 (69.7)	412 (71.7)	241 (71.1)	91 (71.2)	69 (70.0)
Menopausal hormone therapy, ever	447 (44.0)	3	229 (46.5)	158 (40.4)	265 (46.3)	139 (41.0)	60 (45.5)	43 (41.8)
Hormonal intrauterine device, ever	166 (16.6)	18	81 (16.7)	63 (16.3)	75 (13.3)	72 (21.8)	22 (16.9)	19 (18.5)
Age at menarche, years	13 (12–14)	6	13 (12–14)	13 (12–14)	13 (12–14)	13 (12–14)	13.5 (13–14)	13 (13–14)
Nulliparous	122 (12.0)	0	52 (10.6)	54 (13.7)	67 (11.7)	42 (12.4)	16 (12.1)	13 (12.6)
Screening detected (age 45–74 years)	569 (66.2)	159	261 (62.3)	221 (67.8)	317 (63.5)	189 (68.7)	87 (76.3)	63 (74.1)
**Invasive tumor size**		0						
>20 mm (or muscular or skin involvement)	277 (27.2)		149 (30.2)	99 (25.2)	168 (29.2)	82 (24.1)	29 (22.0)	27 (26.2)
**Any axillary lymph node involvement**	389 (38.3)	2	207 (42.1)	139 (35.5)	219 (38.2)	138 (40.6)	43 (32.6)	32 (31.1)
**Receptor status**								
ER^+^	894 (87.9)	1	468 (94.3)	312 (79.6)	491 (85.5)	314 (92.4)	114 (86.4)	89 (86.4)
PR^+^	721 (70.9)	1	376 (76.3)	255 (65.1)	389 (67.8)	253 (74.4)	90 (68.2)	79 (76.7)
HER2 Amplification	110 (11.5)	63	56 (11.9)	37 (9.6)	69 (12.6)	31 (9.3)	17 (17.2)	10 (14.1)
Triple Negative	74 (7.3)	7	10 (2.0)	58 (14.8)	50 (8.7)	19 (5.6)	6 (4.7)	5 (5.1)
**Main histological type**		0						
No special type (formerly ductal)	823 (80.8)		393 (79.7)	337 (85.8)	466 (81.0)	285 (83.8)	93 (70.5)	72 (69.9)
Lobular	117 (11.5)		72 (14.6)	21 (5.3)	72 (12.5)	33 (9.7)	24 (18.2)	12 (11.7)
Other or mixed	78 (7.7)		28 (5.7)	35 (8.9)	37 (6.4)	22 (6.5)	15 (13.4)	19 (18.5)
**Histological grade**		1						
I	256 (25.2)		125 (25.4)	89 (22.7)	126 (21.9)	96 (28.3)	42 (32.1)	34 (33.0)
II	504 (49.6)		267 (54.2)	174 (44.3)	295 (51.3)	166 (49.0)	63 (48.1)	43 (41.8)
III	257 (25.3)		101 (20.5)	130 (33.1)	154 (26.8)	77 (22.7)	26 (19.9)	26 (25.2)
**Ever treatment by last follow-up prior to any event**								
Chemotherapy	259 (25.4)	0	117 (23.7)	113 (28.8)	148 (25.7)	87 (25.6)	29 (22.0)	24 (23.3)
Radiotherapy	644 (63.3)	0	318 (64.5)	253 (64.4)	359 (62.4)	228 (67.1)	73 (55.3)	57 (55.3)
Herceptin	73 (7.2)	0	38 (7.7)	22 (5.6)	46 (8.0)	19 (5.6)	13 (9.9)	8 (7.8)
**ER^+^tumors**								
Tamoxifen	572 (64.0)	0	315 (67.3)	195 (62.5)	318 (64.8)	201 (64.0)	62 (54.4)	53 (59.6)
Aromatase inhibitor	371 (41.5)	0	211 (45.1)	119 (38.1)	223 (45.4)	119 (36.0)	41 (36.0)	35 (39.3)

### Tumor characteristics in relation to CAV1 levels

Positive cytoplasmic CAV1 was associated with several unfavorable tumor characteristics: ER^–^, PR^–^, TNBC, histological grade III, and lower frequency of lobular-type tumors (all *P*_adj_≤0.002) but was inversely associated with ALNI (*P*_adj_ = 0.036). Conversely, strong stromal CAV1 was associated with several favorable tumor characteristics: ER^+^ (*P*_adj_ = 0.002), non-TNBC (*P*_adj_ = 0.048), and lower frequency of histological grade III (*P*_adj_ = 0.002). For combined CAV1 status, the positive/not strong group was associated with unfavorable tumor characteristics (ER^–^, PR^–^, TNBC, histological grade III, and lower frequency of lobular-type tumors; all *P*_adj_ ≤ 0.001).

### CAV1 signaling pathways in TCGA, STRING, and GOBO

**Fig. 2:**
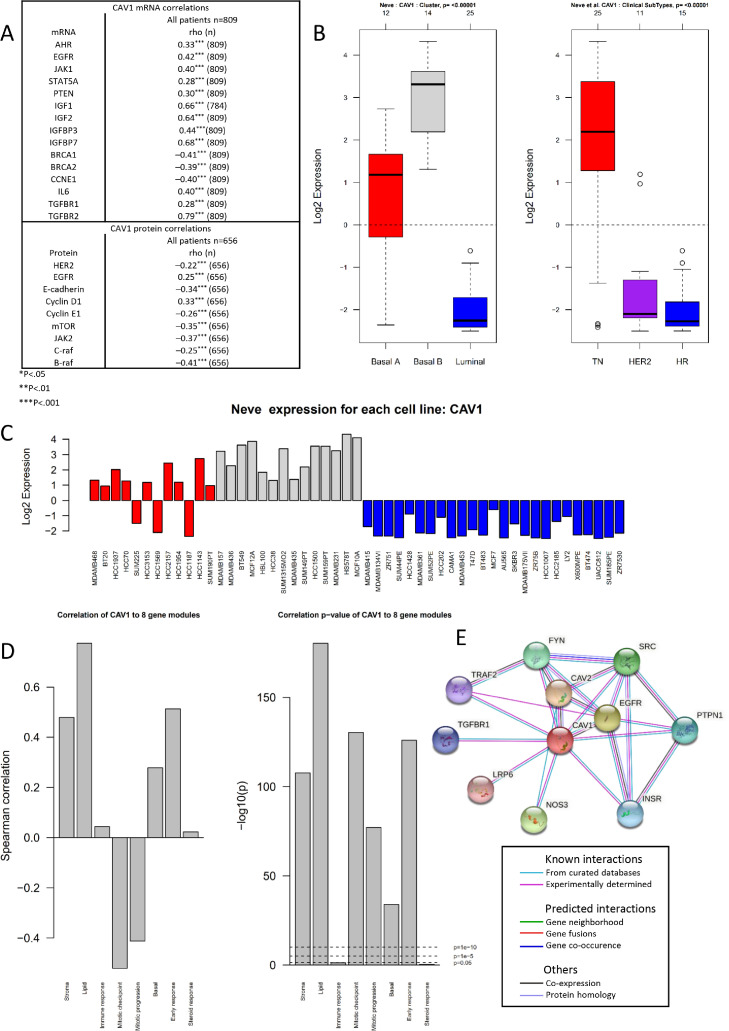
*CAV1* mRNA expression in primary breast cancer cell lines and tumors. **(A)** CAV1 correlations with R_s_ ≥ 0.3 in a subset of 809 breast cancers from TCGA. **(B)** Boxplots of *CAV1* expression across subtypes as defined by Neve et al. [30]. and according to receptor status. **(C)***CAV1* expression (log2) across 51 individual breast cancer cell lines grouped according to Basal A (red), Basal B (gray), and Luminal (Blue) as defined by Neve et al. [30]. (**D**) *CAV1* correlation within 1 881 breast tumors with eight gene modules (Stroma, Lipid, Immune response, mitotic checkpoint, mitotic progression, basal, early response, and steroid response) and corresponding correlation *P*-values from GOBO [28,29]. **(E)** STRING network analysis of the closest functional biological associations for CAV1 [30].

*CAV1* mRNA and protein levels were correlated (R_s_ = 0.47) in TCGA. Both *CAV1* mRNA and protein levels were positively correlated with genes and proteins associated with non-luminal subtypes (particularly basal), cell cycle control, inflammation, EMT, and the IGF/Insulin system, with correlations (R_s_ ≥ 0.3; Fig. 2A). Analyzes in GOBO yielded similar results; *CAV1* was associated with non-luminal subtypes (Fig. 2B,C). In GOBO, *CAV1* mRNA was positively associated with gene modules related to lipid metabolism, stroma interactions, early response to growth factors, and basal pathways while being negatively associated with mitotic regulation (Fig. 2D). Functional networks in STRING showed CAV1’s strong associations with tyrosine kinases, inflammatory markers, TGFβ pathway/EMT, and IGF/Insulin system (Fig. 2E).

### CAV1 levels and prognosis

In the BCblood cohort, the patients were followed for up to 15 years. The median follow-up for the 668 patients still at risk was 9.0 years (interquartile range 7.0–11.1 years). There were 184 patients with any recurrence during the follow-up (116 with distant metastasis). One-hundred-seventy-six patients died during follow-up, of which 96 had a prior recurrence.

The hazards were proportional for cytoplasmic, stromal, and combined CAV1 status for the three main endpoints (all *P_s_* ≥ 0.1). Positive cytoplasmic CAV1 was weakly associated with higher recurrence risk in the univariable analysis but not after adjustment (Supplementary Fig. 1; Supplementary Table 2). Further, cytoplasmic CAV1 was not associated with DMFI. However, positive cytoplasmic CAV1 conferred a borderline lower risk of death in the multivariate analyzes (Supplementary Table 2). Neither stromal nor combined CAV1 status was associated with BCFI, DMFI, or OS, neither in the univariable nor multivariable analyzes.

**Fig. 3:**
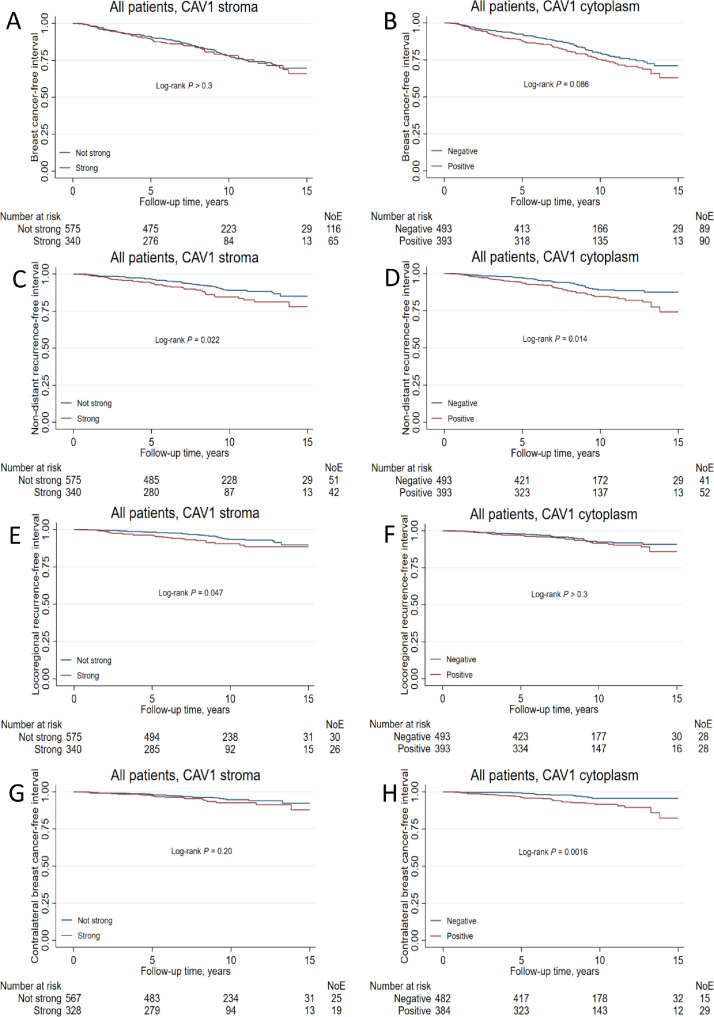
Kaplan-Meier estimates of (**A, B**) breast cancer-free interval (**C, D**) non-distant recurrence-free interval, (**E, F**) distant metastasis-free interval, and (**G, H**) contralateral breast cancer-free interval in relation to CAV1 stromal and cytoplasmic status in all patients. The number of patients is indicated at each follow-up. The study is ongoing; thus, the number of patients decreases with each follow-up.

The increased recurrence risk with positive cytoplasmic CAV1 appeared to be driven by non-distant metastasis. Further survival analyzes with NDRFI, CBCFI, and LRFI as outcomes were conducted for both cytoplasmic and stromal CAV1. Positive cytoplasmic CAV1 conferred over 2-fold risk for contralateral breast cancer HR_adj_ 2.63 (95% CI 1.36–5.10), while stromal CAV1 conferred nearly 2-fold risk for locoregional recurrence HR_adj_ 1.88 (95% CI 1.09–3.24, Fig. 3; Supplementary Table 3).

### Effect modifications by clinicopathological factors on the associations between CAV1 and prognosis

**Fig. 4:**
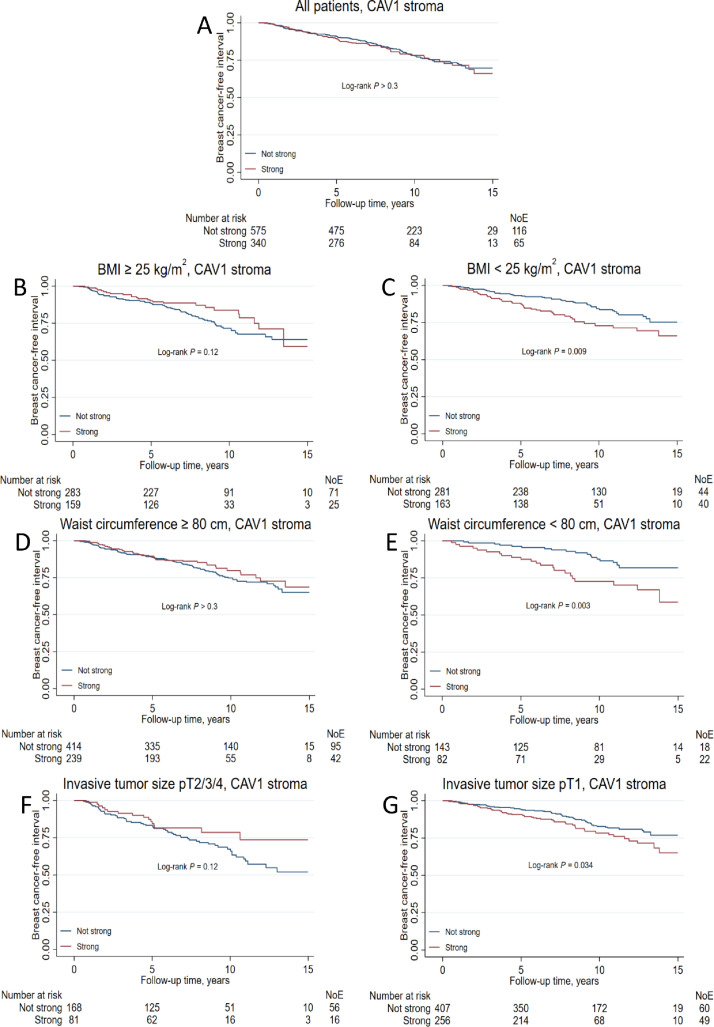
Kaplan-Meier estimates of breast cancer-free interval in (**A**) all patients, (**B, C**) stratified by BMI ≥ 25 kg/m^2^, (**D, E**) stratified by waist ≥80 cm, and (**F, G**) stratified by invasive tumor size treatment in relation to stromal CAV1 status. The study is ongoing; thus, the number of patients decreases with each follow-up.

The impact of strong stromal CAV1 on BCFI was modified by several prognostic factors, BMI ≥25 kg/m^2^ (*P*_interaction_ = 0.002), waist ≥80 cm (*P*_interaction_ = 0.005), and invasive tumor size, pT2/3/4 (*P*_interaction_ = 0.028; Fig. 4). In normal-weight patients, strong stromal CAV1 increased recurrence risk HR_adj_ 1.92 (95% CI 1.22–3.02) but not in overweight patients. Similarly, in patients with small waists (<80 cm), stromal CAV1 increased recurrence risk HR_adj_ 2.25 (95% CI 1.16–4.36) but not in patients with larger waists. Also, in patients with small tumors (pT1), strong stromal CAV1 was associated with increased recurrence risk HR_adj_ 1.61 (95% CI 1.09–2.38) but not in patients with larger tumors. The results indicate that strong stromal CAV1 is associated with increased recurrence risk among low-risk patients (Table 2).

**Table 2:** Multivariable models of CAV1 levels in relation to recurrences in all patients and stratified by BMI, waist circumference, and invasive tumor size.

	Breast cancer event			
	HR		(95% CI)	
CAV1 stroma strong	1.26		0.92–1.73	
TBSAS	1.10		1.04–1.17	
Age, years	1.00		0.98–1.01	
pT2/3/4	2.16		1.55–3.02	
ALNI	1.43		0.98–2.09	
Grade III	1.76		1.19–2.61	
ER^+^	1.16		0.65–2.05	
Chemotherapy	0.94		0.56–1.59	
Radiotherapy	0.75		0.55–1.03	
Trastuzumab	0.72		0.38–1.39	
Tamoxifen	0.64		0.45–0.90	
Aromatase Inhibitor	0.68		0.46–1.00	
	**BMI** **≥** **25** **kg/m^2^**		**BMI** **<** **25** **kg/m^2^**	
	**HR**	**(95% CI)**	**HR**	**(95% CI)**
CAV1 stroma strong	0.85	0.53–1.37	1.92	1.22–3.02
TBSAS	1.12	1.03–1.22	1.07	0.97–1.17
Age, years	1.01	0.99–1.03	0.98	0.96–1.01
pT2/3/4	1.99	1.29–3.09	2.43	1.40–4.19
ALNI	1.44	0.87–2.38	1.43	0.81–2.53
Grade III	1.46	0.85–2.52	2.44	1.37–4.33
ER^+^	1.06	0.48–2.34	1.18	0.50–2.76
Chemotherapy	1.13	0.58–2.23	0.65	0.29–1.47
Radiotherapy	0.75	0.48–1.15	0.74	0.47–1.18
Trastuzumab	0.93	0.42–2.07	0.51	0.16–1.61
Tamoxifen	0.79	0.48–1.28	0.56	0.34–0.92
Aromatase Inhibitor	0.80	0.48–1.35	0.58	0.31–1.06
	**Waist** **≥** **80 cm**		**Waist** **<** **80 cm**	
	**HR**	**(95% CI)**	**HR**	**(95% CI)**
CAV1 stroma strong	1.02	0.70–1.48	2.25	1.16–4.36
TBSAS	1.12	1.05–1.20	1.07	0.92–1.25
Age, years	1.00	0.98–1.02	0.98	0.95–1.01
pT2/3/4	1.79	1.22–2.61	4.53	2.11–9.73
ALNI	1.48	0.96–2.27	1.79	0.78–4.12
Grade III	1.57	1.01–2.44	3.15	1.34–7.42
ER^+^	0.97	0.52–1.83	7.22	1.07–48.74
Chemotherapy	1.03	0.58–1.85	0.64	0.18–2.33
Radiotherapy	0.71	0.49–1.01	0.78	0.40–1.55
Trastuzumab	0.89	0.45–1.76	No event	
Tamoxifen	0.60	0.40–0.90	0.60	0.29–1.26
Aromatase Inhibitor	0.72	0.46–1.12	0.40	0.16–1.02
	**pT2/3/4**		**pT1**	
	**HR**	**(95% CI)**	**HR**	**(95% CI)**
CAV1 stroma strong	0.75	0.41–1.35	1.61	1.09–2.38
TBSAS	1.08	0.98–1.19	1.10	1.02–1.19
Age, years	1.00	0.97–1.03	1.00	0.97–1.02
ALNI	1.19	0.65–2.18	1.61	1.00–2.60
Grade III	1.32	0.74–2.36	2.33	1.37–3.96
ER^+^	0.89	0.33–2.42	1.42	0.65–3.10
Chemotherapy	1.26	0.59–2.69	0.67	0.31–1.44
Radiotherapy	0.96	0.57–1.61	0.68	0.46–1.01
Trastuzumab	0.56	0.23–1.35	1.14	0.41–3.15
Tamoxifen	0.85	0.42–1.71	0.53	0.35–0.82
Aromatase Inhibitor	0.56	0.29–1.10	0.83	0.50–1.38

Missing data for four patients for at least one variable.

TBSAS - Time between surgery and staining.

Moreover, the impact of stromal and cytoplasmic CAV1 on DMFI was modified by TNBC (*P*_interaction_ = 0.013) and HER2 status (*P*_interaction_ = 0.034). There were also interactions between combined CAV1 status and tamoxifen-treatment in patients with ER^+^tumors with regards to both DMFI (*P*_interaction_ = 0.022) and OS (*P*_interaction_ = 0.005).

In the analyzes with multiple imputation and further adjustments for HER2 and BMI, the results remained essentially the same, except for the interaction between tamoxifen and combined CAV1 status for which the interaction became weaker (*P*_interaction_=0.081).

## Discussion

We found that the prognostic impact of CAV1 was highly dependent on anthropometric factors associated with a poor metabolic profile and tumor characteristics. Strong stromal CAV1 was associated with a substantially worse prognosis only in patients with low BMI, small waist, and small tumors, factors that indicate low recurrence risk [35], [36], [37]. It has been shown that upregulation of CAV1 expression in stroma contributes to metastasis and invasion [17]; and that CAV1 is involved in cellular regulation and inflammation, lipid metabolism, and EMT through various pathways [1], [2], [3], which we could also confirm in TCGA, STRING, and GOBO. These pathways are considered markers of an active TME [6].

Moreover, CAV1 protein levels in TCGA were associated with two reactive breast cancer subgroups with an activated TME [14]. We hypothesize stromal CAV1 to be a marker of activated TME with a larger role in metastasis of less aggressive tumors. Larger body sizes have been associated with more aggressive tumor characteristics [24,38]. If so, this could explain the interactions between stromal CAV1 and anthropometric factors or tumor size, where a higher recurrence risk was observed in low-risk patients. Interestingly, *in vitro* and *in vivo* studies have shown that statins inhibit and lower CAV1 levels, hindering its oncogenic role [39,40]. A recent study reported post-diagnostic statin use to protect against distant but not locoregional recurrences [41], but CAV1’s role in this setting is unknown.

CAV1’s role in hypoxia might contribute to the observed increased locoregional recurrence risk associated with strong stromal CAV1[[3], [40], [42]]. Hypoxia decreases the efficacy of radiotherapy used to achieve local control [43]. *In vitro*, hypoxia-induced factor 1 (HIF1) elevated CAV1 levels increased invasiveness through the NF-κB pathway [44]. CAV1 has been associated with radioresistance in both lung and prostate cancer [45,46].

Cytoplasmic CAV1 was associated with a more malignant tumor phenotype, including a strong association with ER negativity, which is consistent with previous studies [11,12,18,19]. We found CAV1 in TCGA and GOBO to be associated with basal subtype, EMT, EGFR expression, and BRCAness, in line with others [18,19,47,48], all common in ER^–^ breast cancer. Despite this, cytoplasmic CAV1 was borderline associated with longer OS but not with overall recurrence risk in multivariable models.

However, cytoplasmic CAV1 was associated with increased contralateral breast cancer risk. Provided the association between higher CAV1 and BRCA1 deficiency or basal phenotype [18,19,47], that are both risk factors for contralateral breast cancer [49], cytoplasmic CAV1 may be a proxy marker for these factors, representing a tumor phenotype that tends to occur in the contralateral breast. The role of CAV1 in contralateral breast cancer merits further study as there are few established risk factors [49] used to tailor preventative measures for patients at increased risk for contralateral disease.

Interestingly, CAV1 has been shown to modulate both trastuzumab uptake and HER2 expression *in vitro* [25,40]. We found effect modifications between HER2 and cytoplasmic CAV1 but also TNBC and stromal CAV1 on distant metastasis-risk. Unfortunately, the HER2^+^ and TNBC subgroups were too small to adequately assess the CAV1’s impact on prognosis. Further investigation in larger HER2^+^ and TNBC cohorts is warranted to assess the prognostic impact of CAV1 in these subgroups. The impact of combined CAV1 status on overall survival was modified by tamoxifen-treatment. Others reported CAV1 co-expression with ER to potentiate downstream signaling, decreasing the efficacy of tamoxifen [50], and potentially explaining our findings. The crosstalk between CAV1 and non-genomic rapid ER signaling may also contribute to tamoxifen-resistance [20].

This study has several strengths, incorporating reliable clinicopathological and anthropometric data combined with tumor tissue from patients in a large population-based cohort considered representative for its catchment area [22]. Furthermore, quality-controlled data from TCGA, GOBO, and STRING databases were used [14,28,29,31]. The antibody used for IHC staining has been previously validated [42]. To date, there is no standardized way of assessing CAV1 IHC staining, reducing comparability between studies. The CAV1 staining was deemed to be homogenous, as previously reported [12,13].

In conclusion, the prognostic impact of CAV1 was highly dependent on its localization, anthropometric, and tumor factors. Stromal CAV1 predicted high recurrence risk in a group of supposedly ‘low-risk’ patients. In all patients, stromal CAV1 also doubled locoregional recurrence risk. Cytoplasmic CAV1 harbors potential as a new predictive marker for metachronous contralateral disease. If confirmed, CAV1 could be used as treatment target and for further risk-stratification.

## Funding

The Swedish Cancer Society (CAN 20 0763), the Faculty of Medicine at Lund University, the Mrs Berta Kamprad Foundation, the South Swedish Health Care Region (Region Skåne ALF 40,620), and the Skåne University Hospital fund. AB holds a young researcher award from ALF (Region Skåne). HT was funded by Region Skåne ST-ALF. The funders had no role in study design and conduct of the study, data collection and analysis, data interpretation, or manuscript preparation and decision to submit the manuscript for publication.

## Data availability

TCGA data can be downloaded from https://portal.gdc.cancer.gov. Data from GOBO is available at http://co.bmc.lu.se/gobo/ and data from STRING is available at https://string-db.org. Clinical data is not publicly available due to privacy laws. Questions regarding data can be directed to the corresponding author.

## CRediT authorship contribution statement

**Christopher Godina:** Conceptualization, Data curation, Formal analysis, Methodology, Software, Validation, Visualization, Writing – original draft, Writing – review & editing. **Vineesh Indira Chandran:** Investigation, Writing – review & editing. **Magdalena Barbachowska:** Investigation, Resources, Writing – review & editing. **Helga Tryggvadottir:** Data curation, Resources, Writing – review & editing. **Björn Nodin:** Investigation, Validation, Writing – review & editing. **Edward Visse:** Software, Writing – review & editing. **Signe Borgquist:** Resources, Writing – review & editing. **Karin Jirström:** Investigation, Resources, Validation, Writing – review & editing. **Karolin Isaksson:** Funding acquisition, Resources, Writing – review & editing. **Ana Bosch:** Funding acquisition, Writing – review & editing. **Mattias Belting:** Conceptualization, Writing – review & editing. **Helena Jernström:** Conceptualization, Data curation, Formal analysis, Funding acquisition, Investigation, Methodology, Project administration, Resources, Software, Supervision, Validation, Writing – original draft, Writing – review & editing.

## Declaration of Competing Interest

Ana Bosch is co-founder and board chair for SACRA therapeutics. She has received travel support from Roche, lecture fees from Eli-Lilly and has participated in Advisory Boards for Pfizer and Novartis. The other authors declare that they have no known competing financial interests or personal relationships that could have appeared to influence the work reported in this paper.

## Appendix Supplementary materials

**Supplementary Figure 1.** Kaplan-Meier estimates of (**A, D, G)** breast cancer-free interval, (**B, E, H**) distant metastasis-free interval, and (**C, F, I**) overall survival in relation to CAV1 stromal, cytoplasmic, and combined status in all patients. The number of patients is indicated at each follow-up. The study is ongoing; thus, the number of patients decreases with each follow-up. Image, application 1

Image, application 2

Image, application 3

Image, application 4
