# Supplementary figures and images for: Interplay between Caveolin-1 and body and tumor size affects clinical outcomes in breast cancer
Source: Transl Oncol. 2022 Jun 1;22:101464. doi: 10.1016/j.tranon.2022.101464 (PMC9166433; doi:10.1016/j.tranon.2022.101464)

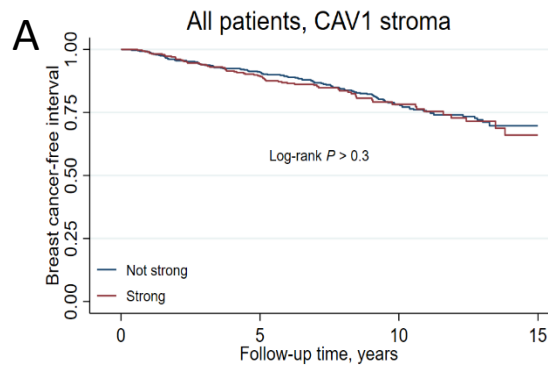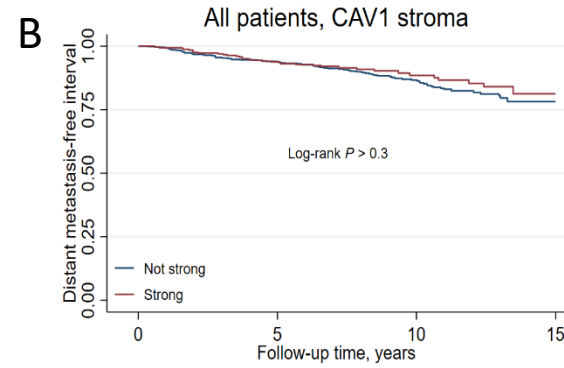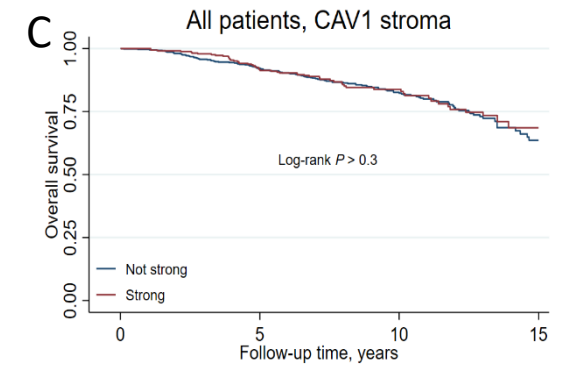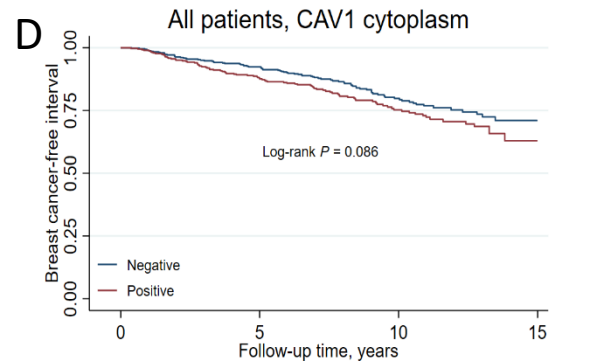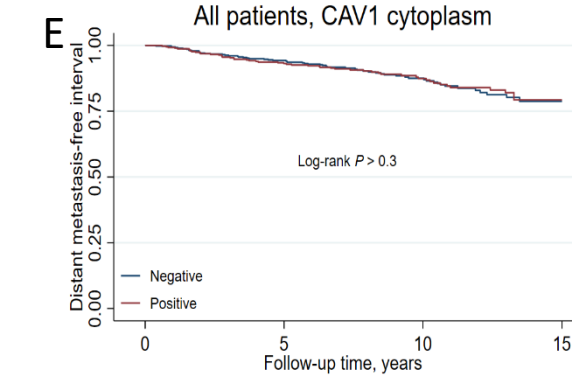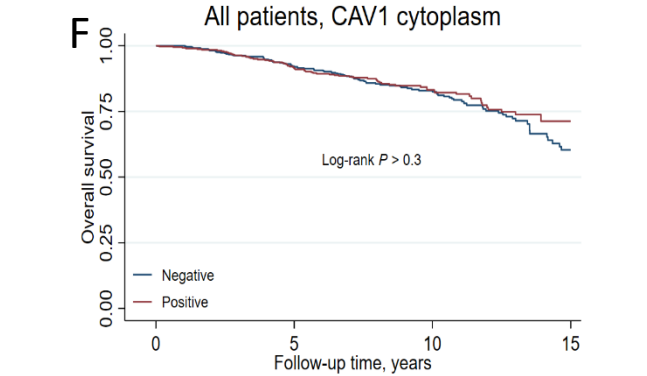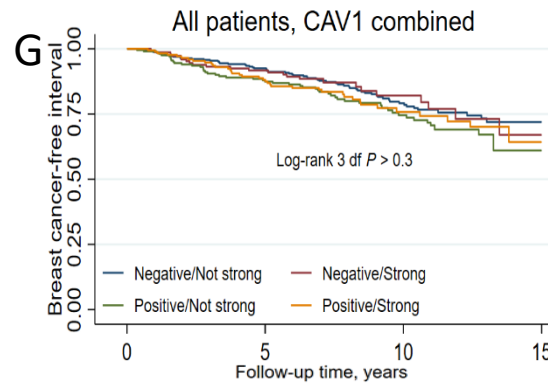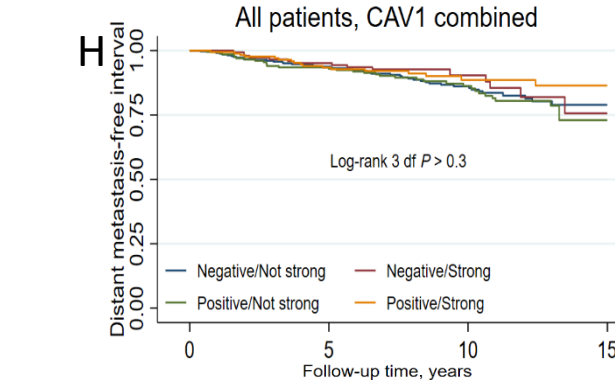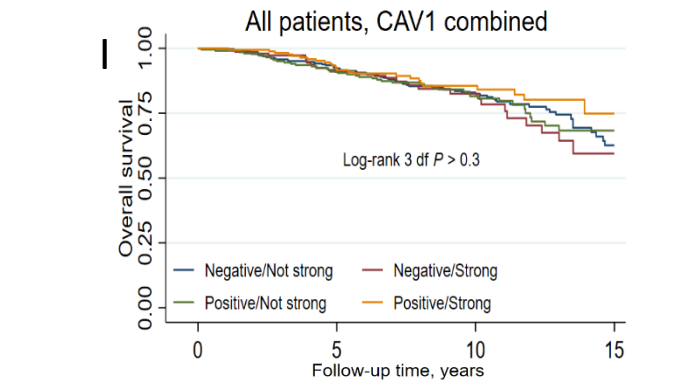

Supplement: Supplementary file 1 — Supplementary Figure 1. Kaplan-Meier estimates of (A, D, G) breast cancer-free interval, (B, E, H) distant metastasis-free interval, and (C, F, I) overall survival in relation to CAV1 stromal, cytoplasmic, and combined status in all patients. The number of patients is indicated at each follow-up. The study is ongoing; thus, the number of patients decreases with each follow-up. [file mmc1.pdf]
