# Supplementary material for: Interplay between Caveolin-1 and body and tumor size affects clinical outcomes in breast cancer
Source: Transl Oncol. 2022 Jun 1;22:101464. doi: 10.1016/j.tranon.2022.101464 (PMC9166433; doi:10.1016/j.tranon.2022.101464)
Supplement: Supplementary file 2 [file mmc2.docx]

| **Supplementary Table 1.** Patient and tumor characteristics in relation to combined CAV1 status | | | | | | | |
| --- | --- | --- | --- | --- | --- | --- | --- |
|  | All | Missing | Combined CAV1 cytoplasm and stroma n=866 | | | | Patients with |
|  | patients |  | Negative/Not strong | Negative/Strong | Positive/Not strong | Positive/Strong | non-evaluable TMAs |
|  | n=1018 |  | n=334 (38.6%) | n=150 (17.3%) | n=207 (23.9%) | n=175 (20.2%) | n=152 |
|  | Number (%) |  | Number (%) | Number (%) | Number (%) | Number (%) | Number (%) |
|  | or Median (IQR) |  | or Median (IQR) | or Median (IQR) | or Median (IQR) | or Median (IQR) | or Median (IQR) |
| Age at inclusion, years | 61.1 (52.1─68.1) | 0 | 61.4 (54.3─68.4) | 58.2 (50.4─67.4) | 62.7 (53.8─68.1) | 59.6 (50.1─67.1) | 62.1 (51.9─69.5) |
| BMI ≥25 kg/m^2^ | 503 (50.8) | 28 | 161 (49.2) | 75 (51.1) | 109 (53.4) | 81 (48.8) | 79 (52.7) |
| Waist circumference ≥80 cm | 731 (74.6) | 38 | 233 (71.9) | 103 (72.0) | 156 (78.4) | 126 (76.4) | 113 (75.8) |
| Breast volume ≥850 ml | 492 (57.3) | 160 | 145 (51.2) | 73 (56.7) | 113 (65.7) | 84 (57.5) | 77 (60.2) |
| Alcohol abstainer, yes | 106 (10.4) | 3 | 32 (9.6) | 20 (13.3) | 20 (9.7) | 20 (11.5) | 14 (9.2) |
| Preoperative smoker, yes | 206 (20.3) | 2 | 67 (20.1) | 29 (19.3) | 37 (17.9) | 37 (21.3) | 36 (23.7) |
| Coffee, ≥2 cups/day | 824 (80.9) | 0 | 274 (82.0) | 111 (74.0) | 178 (86.0) | 139 (79.4) | 122 (80.3) |
| Oral contraceptives, ever | 722 (71.0) | 1 | 241 (72.2) | 107 (71.8) | 147 (71.0) | 122 (69.7) | 105 (69.1) |
| Menopausal hormone therapy, ever | 447 (44.0) | 3 | 162 (48.7) | 62 (41.3) | 86 (41.8) | 70 (40.2) | 67 (44.1) |
| Hormonal intrauterine device, ever | 166 (16.6) | 18 | 45 (13.7) | 36 (24.7) | 30 (14.6) | 32 (18.8) | 23 (15.3) |
| Age at menarche, years | 13.3 (1.4) | 6 | 13 (12─14 | 13 (12─14) | 13 (12─14) | 13 (12─14) | 13 (13─14) |
| Nulliparous | 122 (12.0) | 0 | 34 (10.2) | 17 (11.3) | 29 (14.0) | 23 (13.1) | 19 (12.5) |
| Screening detected (age 45─74 years) | 569 (66.2) | 159 | 177 (60.0) | 81 (69.2) | 116 (67.1) | 99 (67.8) | 96 (75.0) |
| **Invasive tumor size** |  | 0 |  |  |  |  |  |
| >20 mm (or muscular or skin involvement) | 277 (27.2) |  | 99 (29.6) | 45 (30.0) | 63 (30.4) | 33 (18.9) | 37 (24.3) |
| **Any** **axillary lymph node involvement** | 389 (38.3) | 2 | 133 (39.9) | 71 (47.3) | 75 (36.4) | 62 (35.4) | 48 (31.6) |
| **Receptor status** |  |  |  |  |  |  |  |
| ER^+^ | 894 (87.9) | 1 | 313 (93.7) | 146 (97.3) | 148 (71.8) | 155 (88.6) | 132 (86.4) |
| PR^+^ | 721 (70.9) | 1 | 250 (74.9) | 119 (79.3) | 120 (58.3) | 126 (72.0) | 106 (69.7) |
| HER2 Amplification | 110 (11.5) | 63 | 40 (12.7) | 14 (9.5) | 21 (10.4) | 16 (9.2) | 19 (16.4) |
| Triple Negative | 74 (7.3) | 7 | 8 (2.4) | 2 (1.3) | 41 (20.0) | 16 (9.1) | 7 (4.8) |
| **Main histological type** |  | 0 |  |  |  |  |  |
| No special type (formerly ductal) | 823 (80.8) |  | 263 (78.8) | 124 (82.7) | 178 (86.0) | 154 (88.0) | 104 (68.4) |
| Lobular | 117 (11.5) |  | 55 (16.5) | 17 (11.3) | 10 (4.8) | 10 (5.7) | 25 (16.5) |
| Other or mixed | 78 (7.7) |  | 16 (4.8) | 9 (6.0) | 19 (9.2) | 11 (6.3) | 23 (15.1) |
| **Histological grade** |  | 1 |  |  |  |  |  |
| I | 256 (25.2) |  | 76 (22.8) | 47 (31.3) | 41 (19.8) | 46 (26.3) | 46 (30.5) |
| II | 504 (49.6) |  | 193 (57.8) | 71 (47.3) | 84 (40.6) | 86 (49.1) | 70 (46.4) |
| III | 257 (25.3) |  | 65 (19.5) | 42 (21.3) | 82 (39.6) | 43 (24.6) | 35 (23.2) |
| **Ever treatment by last follow-up prior to any event** |  |  |  |  |  |  |  |
| Chemotherapy | 259 (25.4) | 0 | 76 (22.8) | 40 (26.7) | 65 (31.4) | 44 (25.1) | 34 (22.4) |
| Radiotherapy | 644 (63.3) | 0 | 210 (62.9) | 103 (68.7) | 130 (62.8) | 118 (67.4) | 83 (64.6) |
| Herceptin | 73 (7.2) | 0 | 29 (8.7) | 8 (5.3) | 12 (5.8) | 10 (5.7) | 14 (9.2) |
| **ER^+^ tumors** |  |  |  |  |  |  |  |
| Tamoxifen | 572 (64.0) | 0 | 208 (66.5) | 99 (67.8) | 95 (64.2) | 94 (60.7) | 76 (57.6) |
| Aromatase inhibitor | 371 (41.5) | 0 | 150 (47.9) | 56 (38.4) | 64 (43.2) | 54 (34.8) | 47 (35.6) |
