# Supplementary material for: Interplay between Caveolin-1 and body and tumor size affects clinical outcomes in breast cancer
Source: Transl Oncol. 2022 Jun 1;22:101464. doi: 10.1016/j.tranon.2022.101464 (PMC9166433; doi:10.1016/j.tranon.2022.101464)
Supplement: Supplementary file 3 [file mmc3.docx]

| **Supplementary Table 2.** Multivariable Cox regression survival analyses of CAV1 levels in relation to recurrences, distant metastases, and death due to any cause for the entire follow-up period. | | | | | | | | | | | | | | | |
| --- | --- | --- | --- | --- | --- | --- | --- | --- | --- | --- | --- | --- | --- | --- | --- |
| **Breast cancer recurrence** | | | | | | | | | | | | |  | | |
|  | **Total** | **Events** | **Crude*** | | **Adjusted model 1^a^** | | **Adjusted model 1^b^** | | **Adjusted model 2^a^** | | **Adjusted model 2^b^** | |  | |  |
| **CAV1 stroma** | ***n*** | ***n*** | **HR** | **(95% CI)** | **HR** | **(95% CI)** | **HR** | **(95% CI)** | **HR** | **(95% CI)** | **HR** | **(95% CI)** |  |  |  |
| Not strong | 575 | 116 | Ref. |  | Ref. |  | Ref. |  | Ref. |  | Ref. |  |  |  |  |
| Strong | 340 | 65 | 1.12 | 0.82 ─ 1.52 | 1.28 | 0.93 ─ 1.75 | 1.24 | 0.91 ─ 1.70 | 1.26 | 0.92 ─ 1.73 | 1.23 | 0.89 ─ 1.68 |  |  |  |
| **CAV1 cytoplasm** | ***n*** | ***n*** | **HR** |  | **HR** |  | **HR** |  | **HR** |  | **HR** |  |  |  |  |
| Negative | 493 | 89 | Ref. |  | Ref. |  | Ref. |  | Ref. |  | Ref. |  |  |  |  |
| Positive | 393 | 90 | 1.29 | 0.97 ─ 1.74 | 1.24 | 0.91 ─ 1.69 | 1.28 | 0.94 ─ 1.75 | 1.14 | 0.83 ─ 1.56 | 1.18 | 0.86 ─ 1.61 |  |  |  |
| **CAV1 combined** | ***n*** | ***n*** | **HR** |  | **HR** |  | **HR** |  | **HR** |  | **HR** |  |  |  |  |
| Negative/Not strong | 334 | 64 | Ref. |  | Ref. |  | Ref. |  | Ref. |  | Ref. |  |  |  |  |
| Negative/Strong | 150 | 24 | 1.08 | 0.67 ─ 1.73 | 1.04 | 0.64 ─ 1.67 | 1.05 | 0.65 ─ 1.69 | 1.02 | 0.63 ─ 1.64 | 1.02 | 0.63 ─ 1.65 |  |  |  |
| Positive/Not strong | 207 | 50 | 1.35 | 0.93 ─ 1.96 | 1.12 | 0.76 ─ 1.67 | 1.18 | 0.80 ─ 1.76 | 1.05 | 0.70 ─ 1.57 | 1.08 | 0.72 ─ 1.62 |  |  |  |
| Positive/Strong | 175 | 38 | 1.30 | 0.87 ─ 1.94 | 1.45 | 0.96 ─ 2.19 | 1.48 | 0.98 ─ 2.24 | 1.36 | 0.90 ─ 2.07 | 1.40 | 0.92 ─ 2.12 |  |  |  |
| **Distant metastasis** | | | | | | | | | | | | |  | | |
|  | **Total** | **Events** | **Crude*** | | **Adjusted model 1^a^** | | **Adjusted model 1^b^** | | **Adjusted model 2^a^** | | **Adjusted model 2^b^** | |  | |  |
| **CAV1 stroma** | ***n*** | ***n*** | **HR** | **(95% CI)** | **HR** | **(95% CI)** | **HR** | **(95% CI)** | **HR** | **(95% CI)** | **HR** | **(95% CI)** |  |  |  |
| Not strong | 575 | 80 | Ref. |  | Ref. |  | Ref. |  | Ref. |  | Ref. |  |  |  |  |
| Strong | 340 | 35 | 0.86 | 0.58 ─ 1.29 | 1.07 | 0.71 ─ 1.62 | 1.05 | 0.70 ─ 1.58 | 1.06 | 0.70 ─ 1.60 | 1.03 | 0.68 ─ 1.55 |  |  |  |
| **CAV1 cytoplasm** | ***n*** | ***n*** | **HR** |  | **HR** |  | **HR** |  | **HR** |  | **HR** |  |  |  |  |
| Negative | 493 | 62 | Ref. |  | Ref. |  | Ref. |  | Ref. |  | Ref. |  |  |  |  |
| Positive | 393 | 50 | 1.00 | 0.69 ─ 1.45 | 0.93 | 0.62 ─ 1.38 | 0.97 | 0.65 ─ 1.46 | 0.85 | 0.56 ─ 1.28 | 0.89 | 0.59 ─ 1.34 |  |  |  |
| **CAV1 combined** | ***n*** | ***n*** | **HR** |  | **HR** |  | **HR** |  | **HR** |  | **HR** |  |  |  |  |
| Negative/Not strong | 334 | 47 | Ref. |  | Ref. |  | Ref. |  | Ref. |  | Ref. |  |  |  |  |
| Negative/Strong | 150 | 15 | 0.89 | 0.50 ─ 1.61 | 0.83 | 0.46 ─ 1.50 | 0.84 | 0.46 ─ 1.51 | 0.82 | 0.45 ─ 1.48 | 0.83 | 0.46 ─ 1.50 |  |  |  |
| Positive/Not strong | 207 | 32 | 1.12 | 0.72 ─ 1.76 | 0.83 | 0.51 ─ 1.36 | 0.89 | 0.54 ─ 1.47 | 0.77 | 0.46 ─ 1.27 | 0.81 | 0.49 ─ 1.34 |  |  |  |
| Positive/Strong | 175 | 17 | 0.77 | 0.44 ─ 1.34 | 0.97 | 0.55 ─ 1.71 | 1.01 | 0.57 ─ 1.79 | 0.91 | 0.51 ─ 1.62 | 0.96 | 0.54 ─ 1.71 |  |  |  |
| **Death** | | | | | | | | | | | | |  | | |
|  | **Total** | **Events** | **Crude*** | | **Adjusted model 1^a^** | | **Adjusted model 1^b^** | | **Adjusted model 2^a^** | | **Adjusted model 2^b^** | |  | |  |
| **CAV1 stroma** | ***n*** | ***n*** | **HR** | **(95% CI)** | **HR** | **(95% CI)** | **HR** | **(95% CI)** | **HR** | **(95% CI)** | **HR** | **(95% CI)** |  |  |  |
| Not strong | 575 | 115 | Ref. |  | Ref. |  | Ref. |  | Ref. |  | Ref. |  |  |  |  |
| Strong | 340 | 56 | 0.93 | 0.67 ─ 1.29 | 1.20 | 0.86 ─ 1.66 | 1.17 | 0.84 ─ 1.62 | 1.20 | 0.86 ─ 1.68 | 1.19 | 0.86 ─ 1.67 |  |  |  |
| **CAV1 cytoplasm** | ***n*** | ***n*** | **HR** |  | **HR** |  | **HR** |  | **HR** |  | **HR** |  |  |  |  |
| Negative | 493 | 100 | Ref. |  | Ref. |  | Ref. |  | Ref. |  | Ref. |  |  |  |  |
| Positive | 393 | 69 | 0.88 | 0.65 ─ 1.20 | 0.75 | 0.54 ─ 1.05 | 0.75 | 0.54 ─ 1.05 | 0.69 | 0.49 ─ 0.98 | 0.71 | 0.50 ─ 1.00 |  |  |  |
| **CAV1 combined** | ***n*** | ***n*** | **HR** |  | **HR** |  | **HR** |  | **HR** |  | **HR** |  |  |  |  |
| Negative/Not strong | 334 | 69 | Ref. |  | Ref. |  | Ref. |  | Ref. |  | Ref. |  |  |  |  |
| Negative/Strong | 150 | 28 | 1.10 | 0.71 ─ 1.72 | 1.27 | 0.81 ─ 2.00 | 1.29 | 0.83 ─ 2.02 | 1.27 | 0.81 ─ 2.00 | 1.29 | 0.82 ─ 2.03 |  |  |  |
| Positive/Not strong | 207 | 42 | 1.04 | 0.70 ─ 1.53 | 0.78 | 0.51 ─ 1.18 | 0.80 | 0.52 ─ 1.21 | 0.71 | 0.46 ─ 1.10 | 0.74 | 0.48 ─ 1.14 |  |  |  |
| Positive/Strong | 175 | 25 | 0.76 | 0.48 ─ 1.20 | 0.85 | 0.53 ─ 1.36 | 0.84 | 0.52 ─ 1.35 | 0.82 | 0.50 ─ 1.32 | 0.83 | 0.51 ─ 1.35 |  |  |  |
| * Stromal and combined CAV1 status are adjusted for time between surgery and staining in all models | | | | | | | | | | | | |  |  |  |
| Adjusted model 1: Age at inclusion, tumor size, nodal status, grade III, and ER status. Missing data for four patients for at least one variable. | | | | | | | | | | | | |  |  | |
| Adjusted model 2: Model 1+ chemotherapy, radiotherapy, trastuzumab, tamoxifen, and aromatase inhibitors. Missing data for four patients for at least one variable. | | | | | | | | | | | | | | | |
| a: complete case analysis | | b: multiple imputation of all covariates and additional adjustment for BMI ≥25 kg/m^2^ and HER2 status | | | | | | | | | | | | | |
