# Supplementary material for: Interplay between Caveolin-1 and body and tumor size affects clinical outcomes in breast cancer
Source: Transl Oncol. 2022 Jun 1;22:101464. doi: 10.1016/j.tranon.2022.101464 (PMC9166433; doi:10.1016/j.tranon.2022.101464)
Supplement: Supplementary file 4 [file mmc4.docx]

| **Supplementary Table 3.** Multivariable Cox regression survival analyses of CAV1 stroma and cytoplasm in relation to different types of recurrences | | | | | | | | | | | | | | | | | | | | | |
| --- | --- | --- | --- | --- | --- | --- | --- | --- | --- | --- | --- | --- | --- | --- | --- | --- | --- | --- | --- | --- | --- |
|  | **Breast cancer event** | | **Non-distant recurrence** | | | **Locoregional recurrence** | | | **Contralateral breast cancer** | | | | |  | | | | | | |  |
|  | **HR** | **(95% CI)** | **HR** | **(95% CI)** | | **HR** | **(95% CI)** | | **HR** | **(95% CI)** | | | |  |  | |  |  |  |  |  |
| CAV1 stroma strong | 1.26 | 0.92 ─ 1.73 | 1.76 | 1.15 ─ 2.70 | | 1.88 | 1.09 ─ 3.24 | | 1.57 | 0.84 ─ 2.92 | | | |  |  | |  |  |  |  |  |
| TBSAS | 1.10 | 1.04 ─ 1.17 | 1.09 | 1.01 ─ 1.19 | | 1.14 | 1.02 ─ 1.27 | | 1.02 | 0.91 ─ 1.15 | | | |  |  | |  |  |  |  |  |
| Age, years | 1.00 | 0.98 ─ 1.01 | 0.99 | 0.97 ─ 1.01 | | 0.97 | 0.94 ─ 0.99 | | 1.01 | 0.98 ─ 1.04 | | | |  |  | |  |  |  |  |  |
| pT2/3/4 | 2.16 | 1.55 ─ 3.02 | 1.19 | 0.71 ─ 2.02 | | 1.03 | 0.50 ─ 2.11 | | 1.45 | 0.71 ─ 2.96 | | | |  |  | |  |  |  |  |  |
| ALNI | 1.43 | 0.98 ─ 2.09 | 1.17 | 0.68 ─ 2.01 | | 0.84 | 0.40 ─ 1.76 | | 1.63 | 0.76 ─ 3.50 | | | |  |  | |  |  |  |  |  |
| Grade III | 1.76 | 1.19 ─ 2.61 | 2.14 | 1.22 ─ 3.77 | | 3.24 | 1.55 ─ 6.77 | | 1.42 | 0.61 ─ 3.29 | | | |  |  | |  |  |  |  |  |
| ER^+^ | 1.16 | 0.65 ─ 2.05 | 1.35 | 0.58 ─ 3.13 | | 1.69 | 0.58 ─ 4.95 | | 0.93 | 0.28 ─ 3.10 | | | |  |  | |  |  |  |  |  |
| Chemotherapy | 0.94 | 0.56 ─ 1.59 | 0.63 | 0.28 ─ 1.43 | | 0.59 | 0.20 ─ 1.71 | | 0.68 | 0.21 ─ 2.18 | | | |  |  | |  |  |  |  |  |
| Radiotherapy | 0.75 | 0.55 ─ 1.03 | 0.56 | 0.36 ─ 0.85 | | 0.58 | 0.33 ─ 0.99 | | 0.53 | 0.29 ─ 1.00 | | | |  |  | |  |  |  |  |  |
| Trastuzumab | 0.72 | 0.38 ─ 1.39 | 0.66 | 0.20 ─ 2.10 | | 0.82 | 0.20 ─ 3.30 | | 0.68 | 0.13 ─ 3.50 | | | |  |  | |  |  |  |  |  |
| Tamoxifen | 0.64 | 0.45 ─ 0.90 | 0.62 | 0.39 ─ 0.98 | | 0.53 | 0.29 ─ 0.97 | | 0.77 | 0.39 ─ 1.52 | | | |  |  | |  |  |  |  |  |
| Aromatase Inhibitor | 0.68 | 0.46 ─ 1.00 | 0.60 | 0.34 ─ 1.04 | | 0.66 | 0.32 ─ 1.37 | | 0.50 | 0.22 ─ 1.15 | | | |  |  | |  |  |  |  |  |
|  | **Breast cancer event** | | **Non-distant recurrence** | | | **Locoregional recurrence** | | | **Contralateral breast cancer** | | | | |  | | | | | | |  |
|  | **HR** | **(95% CI)** | **HR** | | **(95% CI)** | **HR** | | **(95% CI)** | **HR** | | **(95% CI)** | | |  | |  | |  |  |  |  |
| CAV1 cytoplasm positive | 1.14 | 0.83 ─ 1.56 | 1.49 | | 0.97 ─ 2.29 | 1.00 | | 0.58 ─ 1.75 | 2.63 | | 1.36 ─ 5.10 | | |  | |  | |  |  |  |  |
| Age, years | 0.99 | 0.98 ─ 1.01 | 0.98 | | 0.96 ─ 1.01 | 0.96 | | 0.93 ─ 0.99 | 1.01 | | 0.98 ─ 1.04 | | |  | |  | |  |  |  |  |
| pT2/3/4 | 1.99 | 1.42 ─ 2.78 | 1.04 | | 0.62 ─ 1.76 | 0.87 | | 0.42 ─ 1.79 | 1.30 | | 0.64 ─ 2.66 | | |  | |  | |  |  |  |  |
| ALNI | 1.64 | 1.12 ─ 2.40 | 1.43 | | 0.83 ─ 2.44 | 1.07 | | 0.51 ─ 2.22 | 1.78 | | 0.83 ─ 3.81 | | |  | |  | |  |  |  |  |
| Grade III | 1.53 | 1.02 ─ 2.27 | 1.83 | | 1.03 ─ 3.27 | 2.70 | | 1.28 ─ 5.67 | 1.22 | | 0.51 ─ 2.89 | | |  | |  | |  |  |  |  |
| ER^+^ | 0.99 | 0.56 ─ 1.75 | 1.29 | | 0.55 ─ 3.03 | 1.43 | | 0.49 ─ 4.16 | 1.08 | | 0.32 ─ 3.69 | | |  | |  | |  |  |  |  |
| Chemotherapy | 0.77 | 0.46 ─ 1.26 | 0.48 | | 0.22 ─ 1.07 | 0.42 | | 0.15 ─ 1.16 | 0.61 | | 0.19 ─ 1.94 | | |  | |  | |  |  |  |  |
| Radiotherapy | 0.73 | 0.53 ─ 0.99 | 0.53 | | 0.35 ─ 0.81 | 0.59 | | 0.35 ─ 1.02 | 0.48 | | 0.26 ─ 0.90 | | |  | |  | |  |  |  |  |
| Trastuzumab | 0.81 | 0.42 ─ 1.57 | 0.82 | | 0.26 ─ 2.64 | 0.95 | | 0.24 ─ 3.78 | 0.96 | | 0.18 ─ 5.01 | | |  | |  | |  |  |  |  |
| Tamoxifen | 0.65 | 0.46 ─ 0.92 | 0.66 | | 0.42 ─ 1.05 | 0.55 | | 0.30 ─ 1.00 | 0.88 | | 0.44 ─ 1.73 | | |  | |  | |  |  |  |  |
| Aromatase Inhibitor | 0.68 | 0.46 ─ 1.01 | 0.57 | | 0.33 ─ 1.01 | 0.65 | | 0.31 ─ 1.35 | 0.49 | | 0.21 ─ 1.13 | | |  | |  | |  |  |  |  |
| Missing data for four patients for at least one variable in the multivariable models | | | | | | | | | | | |  |  | | | | | |  |  |  |
